# The Utility of Machine Learning Models for Predicting Chemical Contaminants in Drinking Water: Promise, Challenges, and Opportunities

**DOI:** 10.1007/s40572-022-00389-x

**Published:** 2022-12-17

**Authors:** Xindi C. Hu, Mona Dai, Jennifer M. Sun, Elsie M. Sunderland

**Affiliations:** 1 Mathematica, Inc., 505 14Th St, #800, Oakland, CA 94612 USA; 2 Harvard John A. Paulson School of Engineering and Applied Sciences, Harvard University, Cambridge, MA 02138 USA; 3 Department of Environmental Health, Harvard T.H. Chan School of Public Health, Boston, MA 02115 USA

**Keywords:** Heavy metals, Drinking water, Health-based standards, Risk prediction, Machine learning

## Abstract

**Purpose of Review:**

This review aims to better understand the utility of machine learning algorithms for predicting spatial patterns of contaminants in the United States (U.S.) drinking water.

**Recent Findings:**

We found 27 U.S. drinking water studies in the past ten years that used machine learning algorithms to predict water quality. Most studies (42%) developed random forest classification models for groundwater. Continuous models show low predictive power, suggesting that larger datasets and additional predictors are needed. Categorical/classification models for arsenic and nitrate that predict exceedances of pollution thresholds are most common in the literature because of good national scale data coverage and priority as environmental health concerns. Most groundwater data used to develop models were obtained from the United States Geological Survey (USGS) National Water Information System (NWIS). Predictors were similar across contaminants but challenges are posed by the lack of a standard methodology for imputation, pre-processing, and differing availability of data across regions.

**Summary:**

We reviewed 27 articles that focused on seven drinking water contaminants. Good performance metrics were reported for binary models that classified chemical concentrations above a threshold value by finding significant predictors. Classification models are especially useful for assisting in the design of sampling efforts by identifying high-risk areas. Only a few studies have developed continuous models and obtaining good predictive performance for such models is still challenging. Improving continuous models is important for potential future use in epidemiological studies to supplement data gaps in exposure assessments for drinking water contaminants. While significant progress has been made over the past decade, methodological advances are still needed for selecting appropriate model performance metrics and accounting for spatial autocorrelations in data. Finally, improved infrastructure for code and data sharing would spearhead more rapid advances in machine-learning models for drinking water quality.

**Supplementary Information:**

The online version contains supplementary material available at 10.1007/s40572-022-00389-x.

## Introduction

Water is essential for life, yet the future of safe drinking water faces multifaceted challenges: climate change, aging infrastructure, lack of comprehensive monitoring data, and limited time and resources available to local utilities. The United States (U.S.) federal law that aims to ensure the safety of drinking water for the public is the Safe Drinking Water Act (SDWA). Regulatory standards are in place for more than 90 chemicals, but this represents only a small fraction of the chemicals used in commerce (> 80,000) [1]. U.S. water quality standards are derived from risk-based health thresholds or by considering best-available technology [1]. For many drinking water contaminants, there are insufficient data to characterize risk-based standards. More than 40 million Americans rely on private wells rather than public water supplies to obtain their drinking water [2]. Private wells are not subject to the same monitoring and reporting requirements as public water supplies, and these populations often live in rural and under-resourced areas [3].

The availability of drinking water quality data is key to better understanding the human health impacts of drinking water contaminants. Unfortunately, the current monitoring system has significant gaps: it has low coverage for certain segments of the population like private well owners, it is tested infrequently, and monitoring results are often delayed. Universal screening of chemical drinking water contaminants is costly and logistically challenging. In recent years, an increasing number of studies have focused on developing predictive models for drinking water contamination. Such models may eventually allow a more proactive approach in protecting consumers from potential contaminants of health concern.

Spatial modeling approaches have been successfully used to predict inorganic contaminant concentrations (especially arsenic and nitrate) in well water at the local, regional, and national scales [4•, 5–7, 8••]. Modeling efforts have been motivated by concerns that private well users are not protected by current federal and state regulations. These efforts have been enabled by decades of monitoring data collected by the states to ensure compliance with the SDWA. Recently, modeling efforts have focused on supporting federal regulations for poly- and perfluoroalkyl substances (PFAS) given their priority as drinking water contaminants. For example, predictive models using Bayesian networks and random forest models have been developed to predict PFAS concentrations in private wells in North Carolina and New Hampshire [9•, 10•]. These studies have identified potentially important predictors based on the sources and transport of chemical contaminants in groundwater. Beyond private wells, a similar approach has been developed for community water supplies with expanded predictor lists that consider both natural processes governing the fate and transport of pollution and infrastructure related to the facilities [11, 12•].

Predictive models for water quality can help to prioritize testing in regions that are most likely to have elevated levels of contamination and to better understand factors driving spatial patterns in water quality. As these models improve with expanding monitoring data and refined machine-learning algorithms, they may also be useful for providing exposure predictions for contaminants from drinking water (continuous models). Such predictive exposure surfaces would enable a stronger link between water quality and human health, which would strengthen the impetus for new and stronger water quality regulations where needed. Because the SDWA is a federal law, modeling studies that synthesize regional data to provide national perspectives on the occurrence and magnitude of drinking water contaminants are especially useful.

This review synthesizes studies published in the past ten years that employ predictive analytics and machine learning to model drinking water contamination in the U.S. We conducted a systematic review to search and select studies to be included in this review. Based on this analysis, we summarize the strengths and limitations of existing studies, identify best practices that could accelerate research in this field, and discuss how to better leverage predictive analytics to improve drinking water safety and public health.

## Methods

We searched for all English articles in three databases: National Library of Medicine’s PubMed/MedLine, Elsevier’s EMBASE, and Web of Science Core Collection (including the Science Citation Index and Conference Proceedings Citation Index- Science). We conducted a title and abstract search of the databases on January 20, 2022 for all articles published between January 1, 2012 and January 20, 2022 to capture a 10-year window. We constructed the search terms to capture three main concepts related to machine learning, drinking water, and chemicals. To ensure that search terms were appropriate, we iteratively refined them until we were able to retrieve 20 pre-identified key articles returned by the PubMed search to ensure that all relevant articles would be captured. We excluded articles that were not relevant to our topic of interest through an exclusion (NOT) term to limit the number of total articles returned by each database (Supplementary Information [SI] Table S1). For example, we included in the NOT term “review” to exclude studies that are reviews themselves, “air pollut*” to exclude studies about other environmental media, and “male/female/child” to exclude epidemiological studies. In total, our search returned 1261 articles. After removing over 200 duplicates, we added seven key articles that had not been returned from the original search strategy. We then manually screened titles and abstracts before reading full articles to determine which articles were most relevant for this review (SI Figure S1).

This review includes articles that used machine learning techniques to model chemical water pollutants that occur naturally and/or from anthropogenic sources. We considered articles based on their publication date rather than when data analysis occurred. We excluded articles that did not use a machine learning technique or focused on outcomes other than chemical contaminants (for instance, dissolved oxygen, dissolved organic carbon, and biological contaminants). We also excluded articles that were not in English, analyzed data from outside of the U.S., were an existing literature review (did not report original results), or focused on temporal (seasonal) rather than spatial analysis of chemical concentrations. The selection criteria resulted in a total of 27 articles in our literature review. We extracted and synthesized information from these papers on the machine learning techniques used, predictor categories, model outcomes, and data characteristics (SI Table S2.xlsx).

## Results and Discussion

**Fig. 1:**
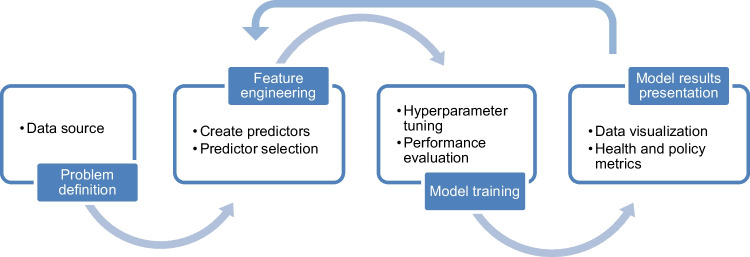
Schematic of a typical workflow used to develop a statistical model for predicting drinking water quality

We synthesized our findings around four common steps used in predictive modeling studies: (1) data sources, (2) feature engineering, (3) model training, and (4) presentation of model results (Fig. 1).

### Data Sources

**Fig. 2:**
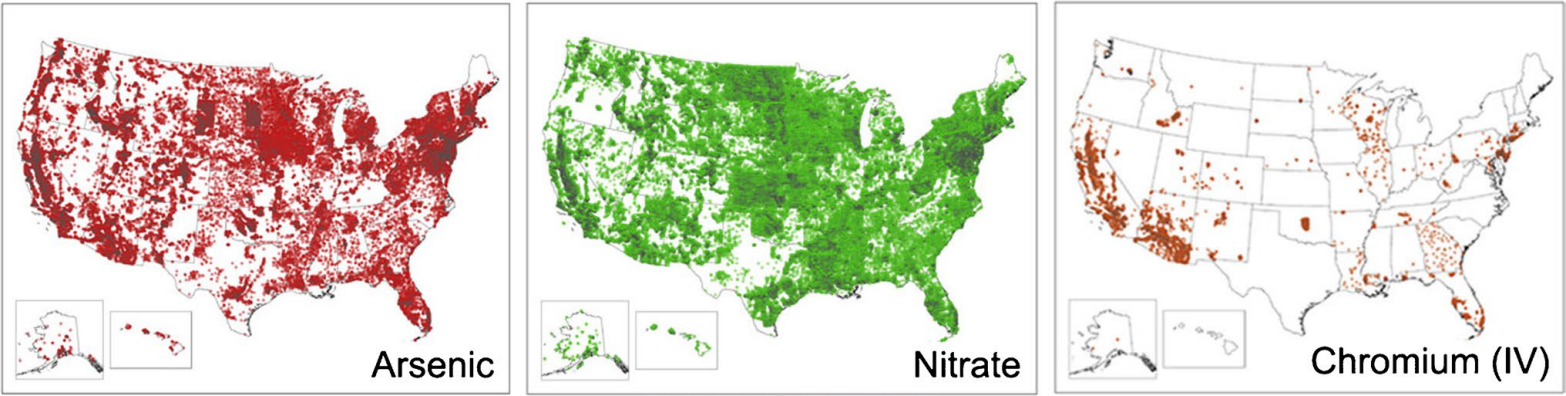
Sampling sites for arsenic, nitrate, and hexavalent chromium well sites downloaded from the Water Quality Portal [13, 14], illustrating differences in data availability across chemicals

Almost half (44%) of the 27 papers reviewed focused on nitrate as a drinking water contaminant and almost 30% were focused on arsenic (Fig. 2). Most studies (67%) used water quality data to develop models at the local scale (at the state-level or smaller). California, in particular the Central Valley, was the most studied locations, considered by six papers in our review.

Large datasets on chemicals in drinking water are publicly available and have utility for machine learning analyses at varying geographic scales (Fig. 2). Most (81%) of the papers included in this review used data from public sources, most commonly from the United States Geologic Survey (USGS) National Water Information System (NWIS) for groundwater concentrations [13]. Other state repositories such as California’s Groundwater Ambient Monitoring and Assessment (GAMA) Program were also frequently cited [15]. Most monitoring databases focused on groundwater rather than public water supplies. Only two of the reviewed studies used the US EPA’s Safe Drinking Water Information System (SDWIS) that contains information on public water supplies [8••, 16]. It is essential that these common data repositories on drinking water are regularly maintained and updated. Table 1 provides examples of popular datasets for groundwater wells and drinking water supplies. Curation of state-level datasets into national datasets would allow for better understanding of national rather than regional scale drinking water quality and would better support federal regulatory efforts. For example, the Water Quality Portal (WQP) (i.e., Fig. 2) was established in 2017 to integrate data from multiple federal, state, tribal, and local agencies into one online database with over 290 million records [17]. The WQP is one of the largest data repositories, combining data from the frequently used USGS NWIS with other datasets to improve public access [17]. Public datasets enable transparency and access for the general public to engage with scientific information. While some datasets are kept private for security reasons, providing aggregated datasets would be useful for future assessments. Other nonprofit organizations such as the Environmental Working Group have curated their own historical databases to explore drinking water contamination [18].

**Table 1:** Public data sources for machine learning modeling related to chemical concentrations in private well, public water supply, or both

Data category		Limitations	Data administrator	Geographic coverage	Example sources
Groundwater wells and relevant predictors					
Contaminant concentrations		Varying data availability depending on chemical and/or regulation, unreported detection limit, high proportion of censored data varying spatial & temporal coverage, ambiguity between well types	US government	National	USGS National Water Information System (NWIS)* [13]; US EPA Storage and Retrieval (STORET) database* [19]
			State government	State	California Groundwater Ambient Monitoring and Assessment (GAMA) [15]; Iowa Statewide Rural Well Water Survey [20]; Minnesota Environmental Mapper & Well Index [21, 22]; New Hampshire PFAS Sampling Map [23]; North Carolina Dry-Cleaning Solvent Cleanup Act Program [24]; North Carolina Department of Environmental Quality [10•]; Florida Well Surveillance Program [25]; Wisconsin Groundwater Retrieval Network (GRN) [26]
			University	Regional	Heidelberg University National Center for Water Quality Research (NCWQR) [27]; University of California, Davis Dairy Monitoring Program [28]; University of Wisconsin-Stevens Point Well Water Viewer [29]
Well characteristics (e.g., location, depth, co-contaminants, chemistry)		Varying spatial & temporal coverage	US government	National	USGS National Water Information System (NWIS)* [13]; US EPA Storage and Retrieval (STORET) database* [19]
			State government	State	California Groundwater Ambient Monitoring and Assessment (GAMA) [15]; Wisconsin Groundwater Retrieval Network (GRN) [26]
			University	Regional	Heidelberg University National Center for Water Quality Research (NCWQR) [27]
Soil characteristics		Varying spatial & temporal coverage	US government	National	USGS Soil Survey Geographic Database (SSURGO) [30]
			State government	State	USDA National Resources Conservation Service US General Soil Map by State (STATSGO2) [31]
Hydrology (e.g., groundwater discharge, streamflow)		Varying spatial & temporal coverage	US government	National	USGS National Water Quality Assessment Program [32]; USGS Ground Water Atlas Map [33]
Water chemistry		Monitors precipitation chemistry, not water sources directly	University	National	University of Wisconsin-Madison National Atmospheric Deposition Program [34]
Geology		Multiple bedrock layers make geological representation difficult	US government	National	USGS National Geologic Map [35]
Aquifer		Multiple bedrock layers make estimation of aquifer types difficult	US government	National	USGS Ground Water Atlas Map [33]
Public water supplies and relevant predictors					
Contaminant concentration		Varying data availability depending on chemical and/or regulations varying spatial & temporal coverage	US government	National	US EPA Unregulated Contaminants Monitoring Report (UCMR) [36]
			Nonprofit	National	Environmental Working Group (EWG) Tap Water Database [37]
Safe Drinking Water Act (SDWA) violations		Varying temporal coverage, limited availability of data downloads	US government	National	US EPA Safe Drinking Water Information System (SDWIS) [38]; US EPA Enforcement and Compliance History Online (ECHO) [39]
			State government	State	California Drinking Water Watch [40]
Public water supply parameters (e.g., source, population served, treatment technology)		Lacking detailed parameters, lacking data dictionary, varying temporal coverage, limited downloading available	US government	National	US EPA Safe Drinking Water Information System (SDWIS) [38]
			State government	State	California Drinking Water Watch [40]
Public water supply service area		Inconsistent, independent reporting & estimation methods between governing entities; lacking unified database	State government	State	Arizona Department of Water Resources [41]; Arkansas Department of Health [42]; California Water Resources Control Board [43]; Connecticut Department of Public Health [44]; Illinois Geospatial Data [45]; Kansas Department of Health and Environment [46]; Massachusetts Department of Environmental Protection [47]; Minnesota Health Department [48]; Missouri Department of Natural Resources [49]; New Jersey Department of Environmental Protection [50]; Oklahoma Water Resources Board [51]; Pennsylvania Department of Environmental Protection [52]; Texas Water Development Board [53]; Utah Division of Drinking Water [54]; Washington Department of Health [55]
			University	Local	Los Angeles Water Hub [56]
General predictors					
Land use (e.g., agriculture, chemical application)		Land use categories could be more generalizable	US government	National	USGS National Land Cover Database (NLCD) [57]
			State government	State	California Land Use Surveys [58]
			University	National	NASA Socioeconomic Data and Applications Center (SEDAC) & Columbia University Center for International Earth Science Information Network (CIESIN) [59]
Potential point source discharge (e.g., industry, military, airport)		Incomplete use and emissions reporting for unregulated contaminant	US government	National	US EPA Permit Compliance System (PCS) & Integrated Compliance Information System (ICIS) [60]; US EPA Toxics Release Inventory (TRI) [61]; US EPA Enforcement and Compliance History Online (ECHO) [39]; US EPA National Priorities List (NPL) [62]; US EPA EJScreen [63]
			State government	State	California GeoTracker [64]; Michigan Environmental Mapper [65]
			University	National	NASA Socioeconomic Data and Applications Center (SEDAC) & Columbia University Center for International Earth Science Information Network (CIESIN) [59]
Land characteristics (e.g., elevation, slope)		Varying spatial resolution compiled over multiple, overlapping data sources	US government	National	USGS National Elevation Dataset [66]
Climate (e.g., temperature, precipitation, evapotranspiration)		Varying spatial resolution requiring potential temporal aggregation	US government	National	Oak Ridge National Lab Daymet [67]
			University	National	PRISM Climate Group, Oregon State University [68]
Sociodemographic		Limited data available depending on spatial unit & year	US government	National	US Census [69]; US EPA EJScreen [63]
			University	National	NASA Socioeconomic Data and Applications Center (SEDAC) & Columbia University Center for International Earth Science Information Network (CIESIN) [59]
Housing characteristics (e.g., housing age, rental status, etc.)		Limited data available depending on spatial unit & year	US government	National	US Census [69]

*database contributing to Water Quality Portal

Some large datasets on drinking water quality such as the Water Quality Portal span a temporal range between the 1960s and present [17]. Metadata on when and where measurements were taken are therefore important in these databases. Most water samples were obtained from the water source (groundwater well) rather than at the point of use. More sample collection at the household level would be useful for understanding contamination at the tap from distribution systems.

Most papers (70%) reviewed in this work developed binary classification models for predicting chemical concentrations beyond a predetermined threshold. By contrast, continuous regression models that could create more detailed prediction maps of areas with poor water quality are lacking. Continuous models were developed for nitrate, tetrachloroethylene (PCE), fluoride, and PFAS and primarily relied on tree-based, neural network, and spatial methods. It is interesting to note that all eight papers that modeled arsenic contamination used binary rather than continuous outcomes. This is an important gap given the substantial public health implications of arsenic contamination in drinking water. Highly censored data (high frequency of non-detect values) makes it challenging for researchers to develop continuous regression models for many drinking water contaminants. Multiple techniques were employed to correct for values below the limit of detection but there was a lack of consistency across studies. Methods included simple imputation, sampling from a modeled distribution, or re-balancing classes using techniques such as oversampling.

Pre-processing of data varied across the studies reviewed and depended on the data source as well as on suitability to the model. Most groundwater studies assigned predictors by well location. Mair and El-Kadi [70] aggregated predictors within a capture zone (spanning multiple wells) due to military sensitivity. Hu et al. [9•] quantified the impact of point sources on private wells using an exponential decay function of hydrological distance between the source and the well location. For atmospheric deposition of PFAS, the authors considered a 10-km buffer radius for estimating source attribution [9•].

Several methods were used across studies to aggregate data if a well was sampled more than once. Most commonly, authors chose to report observed chemical measurements using summary statistics. For example, Ayotte et al. [4•] reported maximum concentration, George and Dixit [71•] reported an average, and Hu et al. [9•] reported median concentrations. Only one paper by Hino et al. [12•] selected randomly among repeated samples. Anning et al. [72] selected a single sample with the greatest ancillary data collected simultaneously. Studies by Rosecrans et al. [16, 73] and Tesoriero et al. [74] selected for samples with known well depth. Erickson et al. [8••] chose to report only the most recent measurements.

These strategies work for each article within the bounds of their data availability. However, what if well locations are unknown or measured predictor values do not exactly match the geographic location of measured outcomes? Aggregating data over an area appears reasonable but requires critical thinking regarding the spatial unit to which the data should be aggregated [70]. The resulting relationships between predictors and outcomes may be different depending on the spatial unit, an issue known as the modifiable areal unit problem (MAUP) [75]. Depending on the research question and data availability, authors should choose the most relevant spatial units depending on the necessary level of detail. Exploring multiple spatial units, such as drawing buffers at multiple radii or focusing on various hydrological unit code (HUC) levels during exploratory analysis of the datasets, can help to decide the best unit of analysis.

### Feature Engineering

Predictor categories were largely consistent among the 27 reviewed papers. While the actual number of factors and specific quantities included in models varied greatly, all models focused primarily on natural factors falling into at least one of the following categories: bedrock geology, hydrology, soil chemistry, and climate. Well construction and groundwater characteristics were also frequently included in most models, along with anthropogenic factors such as land use. These factors have all been previously well-established in the literature and therefore readily accepted as predictors related to chemical contamination of drinking water. The predictor categories overlapped for multiple chemicals. For papers focused on policy or procedural failures (SDWA or inspection violations, for example), the models included characteristics related to community water systems or contamination sources [11, 12•]. The three PFAS papers in this literature review also considered possible point sources in their modeling [9•, 10•, 71•]. In other papers, more discrete model parameters were included. The tetrachloroethylene model by Messier et al. [76] focused only on anthropogenic sources in its land use regression model. The lead (Pb) paper by Fasaee et al. [77] and the PFAS paper by Roostaei et al. [10•] considered household characteristics.

The consistency of these categories demonstrates the availability of these parameters at multiple spatial scales, even for local groundwater flow conditions. However, a more challenging direction may be to expand these parameters from small spatial coverage to large (for instance, national) areas. Most papers (89%) reported variable importance scores and listed top predictors, although the individual predictors were not consistent across the models. For the purpose of making comparisons, it would be helpful if papers reported what broad predictor categories appeared most important rather than the individual predictors themselves. By stating both broad predictor categories as well as specific predictors, variable importance is more easily comparable among papers utilizing similar data characteristics and sources. This may be difficult to quantify because almost all predictor classes were listed among the top predictors for at least one model. Inconsistencies existed among papers in defining and highlighting the top predictors reported by their models. A few papers focused on data availability in choosing wells for their analyses. Poorly described feature maps of environmental factors affecting groundwater quality would impede the ability to credit the predictability of regional models outside their present areas of interest.

### Model Training, Tuning, and Performance

Most models included in these papers performed well (Table 2). Specifically, the accuracy range for binary classification models was between 0.67 and 0.94 (AUC-ROC/C-statistic between 0.72 and 0.92), with over 70% of papers reporting accuracy scores above 0.8. In addition, most papers reported specificity scores greater than sensitivity scores, favoring the correct classification of true negatives. The reported strong model performances (especially for nitrate and arsenic) indicate the potential for machine learning models to similarly execute well for other chemicals that have yet to be explored. Multiclass classification models performed poorer, with the best model from Anning et al. [72] classifying nitrate concentrations correctly 48.6% of the time (although the accuracy increased to 80.4% if the classification was only one category off). For continuous models, large ranges were observed for the most reported metrics: the coefficient of determination (R^2^ = 0.12–0.85) and mean squared error (MSE = 0.05–5.18). However, the most appropriate performance metrics should be carefully considered based on the purpose of the research and characteristics of the input dataset. Some example metrics and their purposes are shown in the table below (Table 2).

**Table 2:** Performance metrics for machine learning models to predict drinking water quality

Performance metric	Purpose	Definition	Limitation	Score range	Papers reported
*Classification*					
Accuracy	Determines proportion of total correct classifications	$\frac{\left(TP + TN\right)}{TP + TN + FP + FN}$ Ranges between 0 and 1	Provides an overoptimistic estimation of the classifier ability on the majority class	Multi-class: 0.367–0.826 Binomial: 0.67–0.94	[4•, 5, 7, 8••, 11, 12•, 72–74, 77–84]
Sensitivity	Determines model’s ability to recall true positives	$\frac{TP}{TP+FN}$ Ranges between 0 and 1	Sensitive to the classification threshold, lower threshold leads to high sensitivity	0.07–0.84	[4•, 5–7, 8••, 12•, 73, 74, 77–79, 81–84]
Specificity	Determines model’s ability to correctly classify true negatives	$\frac{TN}{TN + FP}$ Ranges between 0 and 1	Sensitive to the classification threshold, higher threshold leads to high specificity	0.43–0.98	[4•, 5, 7, 8••, 12•, 22, 23, 27, 28, 30–33]
Area under the receiver operator curve (AUC-ROC) or C-statistic	Determines probability that model will rank randomly chosen positive example higher than randomly chosen negative example	The area under the curve of false positive rate vs true positive rate at different classification thresholds between 0 and 1 Ranges between 0 and 1	Only used for binary classification problem	0.72–0.92	[4•, 7, 8••, 9•, 10•, 12•, 73, 74, 78, 79, 81]
Matthew’s correlation coefficient (MCC)	Measures association between observed & predicted values	$\frac{TP*TN-FP*FN}{\sqrt{\left(TP+FP\right)*\left(TP+FN\right)*\left(TN+FP\right)*\left(TN+FN\right)}}$ Ranges between − 1 and 1	Applies to only one classification threshold	0.31–0.72	[80]
F1 Score	Finds the balance between precision and recall	$\frac{2*TP}{2*TP + FP+FN}$ Ranges between 0 and 1	Applies to only one classification threshold	0.46–0.74	[79, 80]
Cohen’s kappa statistic	Determines how well machine learning classifier matched observations	$\frac{{p}_{0}-{p}_{e}}{1-{p}_{e}}$Ranges between − 1 and 1	Not easy to interpret	0.46–0.62	[7, 8••]
*Regression*					
Coefficient of determination (R^2^)	Determines proportion of variance explainable by predictors	$1-\frac{\sum {\left({y}_{i}-\widehat{{y}_{i}}\right)}^{2}}{\sum {\left({y}_{i}- {\mu }_{y}\right)}^{2}}$ Ranges between 0 and 1	Increases with the number of predictors	0.12–0.85	[4•, 5, 6, 16, 71•, 82, 83, 85••, 86, 87]
Mean square error (MSE)	Measures how spread-out data is around line of best fit	$\frac{1}{n}\sum {\left({y}_{i}-\widehat{{y}_{i}}\right)}^{2}$ Ranges from 0 to ∞	Differs based on the scale of the response variable	0.05–5.18	[16, 76, 80, 83, 85••]
Mean absolute error (MAE)	Measures error between paired observation and prediction	$\frac{1}{n}\sum \left|{y}_{i}-\widehat{{y}_{i}}\right|$ Ranges from 0 to ∞	No penalty for large errors in prediction	0.13–3.06	[80]

*TP*, true positives; *TN*, true negatives; *FP*, false positives; *FN*, false negatives; *p*_0_, overall accuracy of the model; *p*_*e*_, measure of the agreement between the model predictions and the actual class values as if happening by chance

Most papers reported similar metrics to help compare model performance. Most commonly, prediction metrics were reported for hold-out datasets determined either by applying tenfold cross-validation or from pre-specifying a proportion of the total dataset as a randomly chosen test dataset. While some papers (35%) reported both accuracy and AUC-ROC scores, most papers (65%) reported only accuracy scores. Considering only accuracy scores may lead to a biased conclusion regarding model utility, especially when datasets of drinking water contamination are often highly imbalanced. For instance, if data falls into just one bin, the model will replicate the same distribution as the observations regardless of how chemical concentrations are truly distributed. Although the machine learning model will recall the predicted data precisely, these results may not reflect the true distribution of chemical concentrations accurately. In our review, three papers corrected for class imbalance using oversampling techniques: two employed the synthetic minority oversampling technique (SMOTE), and the third article used a spatial declustering method [4•, 80, 82].

### Presentation of Model Results

About two-thirds of the studies (67%) included in this review created surface maps to visualize either the probability of exceeding a threshold with respect to a chemical of interest or for the predicted chemical concentration. Only three papers looked beyond predicting chemical concentrations and calculated additional health and/or policy metrics. Hino et al. [12•] calculated a risk score for community water systems failing inspection. Similarly, Ransom et al. [87] estimated 1.4 million Americans depend on groundwater with nitrate levels exceeding 10 mg/L and Ayotte et al. [4•] estimated that 2.1 million Americans use domestic well water with arsenic values exceeding 10 µg/L. The 18 total papers presenting predicted surface maps may be useful when overlaid with maps related to demographics or national disease burdens. Spatially linking groundwater contamination data with other datasets is critical for connecting drinking water quality concerns to health, environmental restoration, and environmental justice issues. Overlaying this information by zip code or county information would also help to make this information more accessible by the general public since individuals may be more familiar with their residential locations than the physical location of their drinking water supply. However, such an effort would need to take into consideration the MAUP, as discussed above. The MAUP is a source of bias that can present inconsistent statistical results based on the size and shape of the spatial unit analyzed and is especially relevant when aggregating data [75]. To enhance accessibility for a wider audience, a single public repository of downloadable drinking water contamination data accompanied by maps would improve public understanding and transparency of water quality issues. A user-friendly website (for instance, story maps) that walks visitors through general findings in their area of interest would better serve the science communication aspect of these results so that individuals beyond just researchers or water managers can engage with the data. Several efforts in this direction set good examples for how they can be useful for communicating drinking water exposure information to the public but have either been discontinued or limited in scope chemically or spatially [88–91]. Additional work in this area has the potential to inform both communities and researchers interested in managing risks posed by drinking water contaminants.

## Conclusions and Future Research Directions

Big data and machine learning models have been used to predict drinking water contamination for both regulated contaminants such as arsenic and nitrate, as well as emerging chemicals of concern such as PFAS. They show great promise as an alternative and complementary way of assessing drinking water quality compared to traditional grab sample monitoring, which is time and resource intensive and places a considerable burden on regulatory bodies (for community water supplies) and private property owners (for domestic wells). Many existing studies show good model performance for predicting whether drinking water quality exceeds a certain threshold (binary prediction) but models perform more poorly when predicting absolute contamination levels (continuous prediction). Categorical models are best used to enhance traditional sampling schemes for monitoring drinking water quality. Model results are most useful when they are interpreted with the expert knowledge of local conditions, such as verifying susceptible emission sources.

Improving models with continuous outcomes is an important future area for improvement in this field, and is needed to bridge the gap between environmental and human exposures. Better mechanistic understanding of sources of drinking water contaminants, transport, and distribution could be used to develop a comprehensive list of factors influencing these processes that could be included as predictors in such models. A future application of such models includes improved exposure assessment for drinking water contaminants in epidemiologic studies to better understand impacts on human health, following examples for nitrate and arsenic [7, 82].

Other future priorities for research that would aid in establishing drinking water standards at the federal level include developing national scale models that follow the examples created for arsenic and nitrate [4•, 7, 12•, 87]. Availability of monitoring data across the entire country for arsenic and nitrate has made it possible to develop prediction models at the national scale. For emerging contaminants such as PFAS, state-level monitoring datasets exist but additional efforts are needed to synthesize such data into a national scale monitoring data repository. However, when combining datasets of contaminants in drinking water from multiple sources, different reporting limits and detection limits can pose a challenge to data interoperability and needs to be given special considerations. Remaining challenges toward addressing these goals include data availability and interoperability at the national scale, methodological advances in training and evaluating models including choosing the appropriate model performance metrics and accounting for spatial autocorrelation in model training, and better incentives for code sharing to facilitate model averaging for better predictive results.

### Future Research Directions

Improving public data sharing is essential for advancing this field. Inorganic contaminants are the focus of publicly available databases such as the USGS NWIS [13]. For emerging organic contaminants such as PFAS and other unregulated chemicals, most data used to train predictive models are still owned by individual investigators or state agencies. Improved data sharing would enhance collaboration and allow for training better models. Confidentiality concerns represent a barrier toward these goals (i.e., preserving the privacy of private well samples). However, approaches drawn from the health care machine learning literature could provide a potential solution. In the health care field, several methods such as resampling, probabilistic graphical modeling, latent variable identification, and outlier analysis have been proposed to develop synthetic data to preserve patient privacy [92, 93].

Data interoperability that allows available data on contaminant occurrence to be related to environmental and sociodemographic factors is essential. Presently, there are incongruent spatial scales and coverages of training data for different predictors (Table 1). For example, training data and model predictors may be available as vector files with clearly defined boundaries such as public water supply service areas or county boundaries but can be challenging to combine due to differing spatial boundaries. High-resolution raster files such as temperature data from the PRISM climate group [94] and sociodemographic data from the Socioeconomic Data and Applications Center [59] show promise for facilitating spatial data linkages. Another strength of these data sources is that they provide information for broad categories, such as multiple socioeconomic (education attainment, income, poverty level) or climate (precipitation, relative humidity, temperature) variables. Sharing these common input variable sources facilitates easier comparison among papers.

Several methodological improvements would improve the performance of machine-learning models for drinking water quality. First, there is a need for improved techniques for handling imbalanced (highly censored) data during the model training process. If one class of the outcome is rare, the overall accuracy will be biased. Imagine a sample with 98% negatives and 2% positives; a “dumb” classifier that blindly predicts negatives would generate a 98% accuracy score but would be far from a strong model. Instead, focusing on the accuracy per class in the confusion matrix can still be useful for assessing the frequency of the true positive rate and true negative rate. As these predictive models are treated as a type of decision support tool, the exact choice of evaluation metrics will also depend on the use case, such as weighting sensitivity more if the decision makers are worried about false negatives. Other techniques to handle imbalanced data such as downsampling (training on a low subset of the majority class) and upweighting (adding weight to the downsampled class) are also useful and could be more frequently applied. While these are often used in the machine learning literature [95, 96], their appearance in environmental predictive modeling is still rare. Another methodological gap includes how to incorporate spatial autocorrelation into drinking water quality prediction. Failure to account for spatial autocorrelation may result in higher bias in prediction especially when the spatial autocorrelation is very strong, or the predictors included in the model fail to account for the underlying spatial structure. This is an active area of research with different solutions being proposed and no dominant solutions yet [97–99].

Better incentives for code sharing are needed to promote reproducible science and facilitate model averaging. Several journals, such as PLos ONE and Nature, have set expectations that author-generated code underpinning the findings in a manuscript needs to be made publicly available. Model averaging is a technique to reduce modeling uncertainty by making predictions using multiple models that could have promise in this area. Several air pollution and watershed modeling studies have utilized Bayesian Model Averaging and reported that it serves as a strong alternative to model selection as it improved the prediction performance of models in a logical and meaningful way [100, 101].

Safe drinking water is essential for protecting public health. Presently, predictive models are helpful for identifying high-risk areas to prioritize sampling efforts. Although many advances in this field have occurred over the past decade, additional progress is needed for widespread use. Priorities for the future include methodological advances for measuring model performance appropriately and accounting for spatial autocorrelation, and better infrastructure and more resources devoted to data and code sharing.

## Supplementary Information

Below is the link to the electronic supplementary material.

Supplementary file1 (DOCX 47 KB)

Supplementary file2 (XLSX 144 KB)

## Data Availability

All data are available in the main text or the supplementary materials.
