# Supplementary material for: The Utility of Machine Learning Models for Predicting Chemical Contaminants in Drinking Water: Promise, Challenges, and Opportunities
Source: Curr Environ Health Rep. 2022 Dec 17;10(1):45–60. doi: 10.1007/s40572-022-00389-x (PMC9883334; doi:10.1007/s40572-022-00389-x)
Supplement: Supplementary file 1 — Supplementary file1 (DOCX 47 KB) [file 40572_2022_389_MOESM1_ESM.docx]

**Supplementary Information**

**The utility of machine learning models for predicting chemical contaminants in drinking water: promises, challenges and opportunities**

Xindi C. Hu, Mona Dai, Jennifer M. Sun, Elsie M. Sunderland

**Figure S1.** Flow chart of the review from initial literature search to final article selection.

PubMed

n = 475

EMBASE

n = 568

Web of Science

n = 218

Total articles

n = 1,131

Articles passing title screening

n = 337

Key articles

n = 7

Duplicates

n = 136

Articles passing abstract screening

n = 95

US focused articles

n = 27

**Table S1.** Search terms used for literature search conducted on January 20, 2022 returning articles between January 1, 2012 and January 20, 2022

| **Database** | **Number of articles returned** | **Search Terms** |
| --- | --- | --- |
| National Library of Medicine’s PubMed/MedLine | 475 | (("machine learning"[Mesh] OR "data mining"[Mesh] OR "models, statistical"[Mesh] OR "models, chemical"[Mesh] OR "data analysis"[Mesh] OR "Water Pollution, Chemical/statistics and numerical data"[MeSH] OR "machine learning"[tiab] OR "data mining"[tiab] OR (“WATER POLLUTION” [tiab] AND “STATISTICS”[tiab]))  **AND** ("drinking water"[Mesh] OR groundwater[Mesh] OR "water supply"[Mesh] OR "water wells"[Mesh] OR groundwater[tiab] OR "water pollut*"[tiab])  **AND** ("inorganic chemicals"[Mesh] OR "water pollut*"[Mesh] OR pesticides[Mesh] OR nitrates[Mesh] OR fluorid*[Mesh] OR nitrate*[tiab]))  **NOT** (review[pt] OR "air pollut*"[mesh] OR infant*[Mesh] OR child*[Mesh] OR female[Mesh] OR male[Mesh] OR cohort*[Mesh] OR waste*[Mesh] OR DNA[Mesh] OR soil*[Mesh] OR voluntary*[Mesh] OR motion[Mesh] OR minerals[mesh] OR kinetics[mesh] OR spectrometry[mesh] OR carbon*[Mesh] OR petroleum[Mesh] OR "Models, Economic"[Mesh] OR phylogeny[Mesh]) |
| Elsevier’s EMBASE | 568 | (('machine learning'/exp OR 'data mining'/exp OR 'models, statistical'/exp OR 'models, chemical'/exp OR 'data analysis'/exp OR 'Water Pollution, Chemical/statistics and numerical data'/exp OR 'machine learning':ab,ti OR 'data mining':ab,ti)  **AND** ('drinking water'/exp OR ‘groundwater’/exp OR 'water supply'/exp OR 'water wells'/exp OR ‘groundwater’:ab,ti OR 'water pollut*':ab,ti)  **AND** ('inorganic chemicals'/exp OR 'water pollut*'/exp OR ‘pesticides’/exp OR ‘nitrates’/exp OR ‘fluorid*’/exp OR ‘nitrate*’:ab,ti))  **NOT** (‘review’/it OR 'air pollut*'/exp OR ‘infant*’/exp OR ‘child*’/exp OR ‘female’/exp OR ‘male’/exp OR ‘cohort*’/exp OR ‘waste*’/exp OR ‘DNA’/exp OR ‘soil*’/exp OR ‘voluntary*’/exp OR ‘motion’/exp OR ‘minerals’/exp OR ’kinetics’/exp OR ‘spectrometry’/exp OR ‘carbon*’/exp OR ‘petroleum’/exp OR 'Models, Economic'/exp OR ‘phylogeny’/exp) |
| Web of Science Core Collection (including the Science Citation Index and Conference Proceedings Citation Index- Science) | 218 | TS=("machine learning" OR "data mining" OR "statistical model*” OR "data analysis") **AND**  TS = ("drinking water" OR groundwater OR "water supply" OR "water wells" OR “water pollut*”) **AND**  TS = ("inorganic chemicals" OR “water pollut*” OR pesticides OR nitrates OR fluorid*) **NOT**  TS = ("air pollut*" OR infant* OR child* OR female OR male OR cohort* OR waste* OR DNA OR soil* OR voluntary* OR motion OR minerals OR kinetics OR spectrometry OR carbon* OR petroleum OR "Models, Economic" OR phylogeny) |

**Table S2.** Data extraction workbook

See attached Table S2.xlsx
